# What influences UK-practising consultant hip surgeons’ decision-making about implant fixation? An interview study

**DOI:** 10.1136/bmjopen-2026-118009

**Published:** 2026-07-10

**Authors:** Cecily Palmer, Petra Baji, Michael R Whitehouse, Jonathan Thomas Evans, Ashley W Blom, Mike Reed, Elsa M R Marques, Andrew J Moore

**Affiliations:** 1 Musculoskeletal Research Unit, Bristol Medical School, Learning and Research Building Level 1, University of Bristol, Southmead Hospital, Bristol, UK; 2 Department of Public Health and Sports Sciences, University of Exeter, Exeter, UK; 3 Faculty of Biology, Medicine and Health, The University of Manchester, Manchester, UK; 4 Northumbria Healthcare NHS Foundation Trust, North Shields, UK; 5 Health and Community Sciences, University of Exeter, Exeter Medical School, Exeter, UK

**Keywords:** Hip, Clinical Decision-Making, Adult orthopaedics, QUALITATIVE RESEARCH

## Abstract

**Abstract:**

**Objectives:**

To explore the views of UK-based consultant hip surgeons about implant fixation choice and identify factors that inform decision-making about use of cemented, hybrid or uncemented implant fixation when performing total hip replacement in adults aged under 70 years.

**Design:**

Qualitative semistructured interview study. Recruitment was through consultant participation in a national online survey. Purposive sampling was used to ensure a range of implant fixation methods in the final sample. Data were audio recorded, transcribed, anonymised and analysed inductively to generate themes describing influential factors on surgeons’ decision-making about fixation choice.

**Setting:**

UK elective primary total hip arthroplasty. Consultant hip surgeons representing 15 English NHS sites and 2 Scottish Health Boards and one private surgeon took part in a telephone or online interview.

**Participants:**

20 consultant hip surgeons participated, with consultant-level years in practice ranging from 1 to 25+ years. Seven participants primarily used cemented/hybrid constructs and 13 primarily used uncemented/hybrid constructs in patients aged under 70.

**Results:**

Six themes describing influences on surgeons’ decision-making about fixation were identified; ‘patient characteristics’; ‘patient preferences and implant availability’; ‘training and establishing a fixation skillset’; ‘formal evidence of outcomes’, ‘personal outcomes and accumulated clinical experience’ and ‘surgical speed and cost effectiveness as secondary benefits’. All surgeons described patient characteristics that influenced their decision-making, and the sample contained surgeons who preferred cemented, hybrid and uncemented constructs respectively for young, uncomplicated patients. Bone quality was particularly influential and surgeons used age, comorbidities, activity level and sex as indicators of likely bone quality. Poorer bone was agreed to require a cemented stem; however, cup fixation varied, resulting in use of cemented and hybrid constructs when bone was poor. Varied femoral anatomy, including Dorr C, was commonly deemed to require cemented stems within either cemented or hybrid constructs; dysplastic or sclerotic sockets were deemed to require uncemented cups within either uncemented or hybrid constructs. Patient characteristics such as body mass index, likely need for future revision and risk of infection were influential, but not towards any single fixation method. Many surgeons described being trained into geographical/unit fixation traditions, building skill in two fixation methods rather than three. Good outcomes were undetachable from skilled expertise and therefore continued use of specific fixations was reinforced, particularly when surgeons’ personal National Joint Registry outcomes evidenced good performance. Regardless of fixation method preferences, surgeons referred to (at times the same) formal research evidence that supported their fixation choices, as well as their accumulated clinical experience. Increased surgical speed of uncemented components and lower implant cost were not perceived to be key drivers of fixation choice as patient outcomes were prioritised. However, surgical speed was acknowledged as potentially influential in relation to private practice or high volume lists, and costs considered secondarily if good implant outcomes had been established by formal evidence.

**Conclusions:**

Multiple factors exert influence on surgeons’ decision-making about fixation. Community equipoise was identified among UK hip surgeons relating to which fixation method is superior for young, uncomplicated patients or relative to patient characteristics.

**Trial registration number:**

ISRCTN14346605.

**Strengths and limitations of this study**

- Use of qualitative methods has enabled in-depth exploration of the factors that influence hip surgeons’ decision-making about use of cemented, hybrid or uncemented implant fixation in total hip replacement for osteoarthritis.
- The sample size of 20, varied in years of consultant experience, recruited across multiple NHS Trusts and comprising experiences across three fixation methods provided rich textual data and enabled robust analysis and data saturation.
- Recruitment of consultant hip surgeons via participation in an online survey may have resulted in a particularly research-oriented sample of participants, as such responses may have emphasised evidence-based and patient-centred reasoning, potentially understating institutional or habitual factors that influence everyday decision-making.
- Inclusion of only UK-practising consultant surgeons may limit transferability to other healthcare systems where implant availability, procurement processes, and registry processes may differ.

## Background

**Figure 1:**
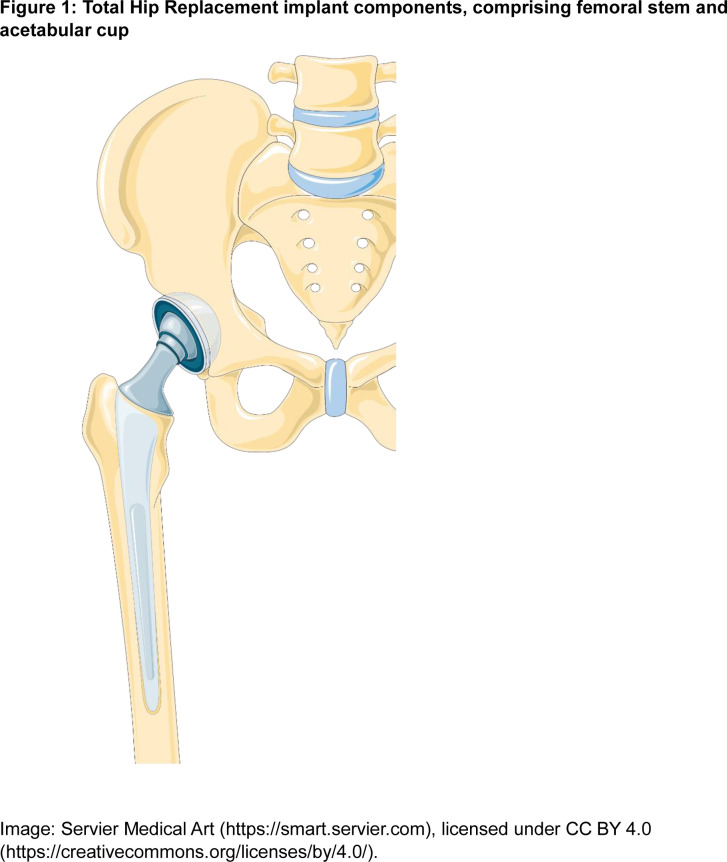
Total hip replacement implant components, comprising femoral stem and acetabular cup.

 Total hip replacement (THR) is one of the most common elective surgeries performed worldwide, most frequently due to osteoarthritis. The joint is then replaced with a femoral stem and an acetabular cup (see figure 1) (Image: Servier Medical Art (https://smart.servier.com), licensed under CC BY 4.0 (https://creativecommons.org/licenses/by/4.0/).) normally with a modular femoral head and overall this collection of implants is referred to as a construct. With multiple manufacturers, in the UK over 300 different combinations of stem and cup bearing materials and fixations are used in primary elective THR. There are different methods by which implants are fixed to the patient’s existing bone.

- Cemented constructs use polymethylmethacrylate cement to attach both the femoral stem and acetabular component to the bone.
- An uncemented (or cementless) construct uses a ‘press-fit’ (sometimes supplemented by screw fixation for additional support) and subsequent osseous integration where living bone grows directly onto the femoral and acetabular components.
- Hybrid constructs contain a cemented femoral stem and an uncemented acetabulum.

The UK-based National Joint Registry (NJR) reports that use of hybrid fixation in primary THR has approximately tripled since 2008 and was used in 42.5% of primary THRs in 2024, followed by uncemented fixation in 39.7% of primary THRs.^1^ Use of cemented fixation has been declining since 2013.^1^

Although three-quarters of NHS patients aged 64 and under receive uncemented or hybrid implants, there is no high-quality randomised controlled trial (RCT)-derived evidence that these constructs have a lower revision rate compared with metal-on-polyethylene fully cemented implants for younger patients or that they have better outcomes in relation to pain or Harris Hip Score; and further they were not cost-effective in a pre-covid economic evaluation.^1^,^5^ However, results from analyses of national registry data (which collect and analyse data relating to arthroplasty surgery) report different results. UK-based NJR data has reported that cemented and hybrid constructs have a lower risk of revision than uncemented constructs at all timepoints after primary hip replacement and that hybrids are superior from 10 years post-primary THR for (men and women in the 55–64 and 65–74 age groups).^1^ Analysis of Nordic Arthroplasty Registry data identified that cemented implants had a higher 10-year survival estimate than uncemented and hybrid implants in patients aged 65–74 and 75 or older but that in patients aged 55–64 cemented and uncemented implants had similar survival estimates.^6^ Analysis of New Zealand joint registry data identified that cemented implants were associated with the lowest revision estimates for all causes in the short term, while uncemented THR had the lowest rate of aseptic loosening in patients under 65 years of age.^7^ When broadening outcomes to consider all reoperations (whether revision of the implant is included or not) for postoperative periprosthetic femoral fracture, recent evidence suggests that collared uncemented stems performed better in men aged 68 and older than cemented stems (used in cemented and hybrid implant constructs).^8 9^ The mismatch between randomised and observational registry evidence, particularly for the younger patients undergoing primary elective THR, highlights the fact that surgeons’ choice of implants for their patients may play an important role in determining patients’ outcomes after surgery. Decision-making in orthopaedic surgery is affected by multiple factors, including formal codified and managerial knowledge, medical socialisation, cultural, normative and political influences, training, formal education and experiential factors.^10 11^ However, to date there has been little empirical investigation into how surgeons make decisions about implant constructs and fixation specifically for THR in younger patients. The Hip Implant Prosthesis Programme for the Younger (HIPPY) Total Hip Replacement patients has designed an RCT (commenced in 2024) to generate high quality evidence to determine clinical and cost effectiveness of cemented, uncemented, and hybrid implant fixations for people aged under 70 requiring primary hip replacement for osteoarthritis.^12^ Within the HIPPY programme, we further explored surgeons’ preferences for implant fixations and reasons informing their choices when deciding on fixations for patients having NHS-funded primary elective THR in the UK. This paper reports the findings of this qualitative study, to best inform the design of the HIPPY RCT and implementation of its findings when results are reported.

## Methods

### Study design

Interpretative research using qualitative methods offers the best way to understand participants’ experiences and beliefs and the influences preferences and contexts involved in their day-to-day practices.^13 14^ We conducted in-depth semistructured interviews with 20 UK-based hip surgeons to explore fixation practices and identify factors that inform decision-making about implant fixation when performing primary THR in adults under 70 years.

#### Inclusion criteria

Consultant level hip surgeon with current or past experience of performing primary elective THR for NHS-funded patients.

#### Exclusion criteria

Unable to provide informed consent.

### Recruitment/sampling

Interview participants were recruited via a UK national online survey of consultant hip surgeons’ fixation practice for THR.^15^ The survey was distributed via the British Hip Society to their mailing list comprising predominantly UK-based consultant hip surgeons, and via social media platform X, and included information about the survey, the wider HIPPY research programme, and the interview study.^16^ In total, 101 hip surgeons completed the survey between January and October 2024, of which 65 indicated their willingness to participate in a one-off qualitative interview (telephone or online) about their fixation decision-making in primary THR. Within the survey, participants were asked to disclose which fixation method they currently ‘most used’ for primary THR patients aged under 70 years. We used purposive sampling to ensure that participants using cemented, uncemented and hybrid implants (not including reverse hybrid) were represented in the final sample. 48 interested surgeons were contacted by email and provided with the interview study information pack, and invited to ask questions, before arranging a convenient time for the interview to take place. Non-participation was primarily due to non-response to invite email/reminders. We aimed to recruit a sample size of 20 surgeons to achieve data saturation (ie, the point at which no new information is identified within subsequent interviews).^17 18^

### Patient and public involvement

The HIPPY research programme of which this study is part has been collaboratively designed with the HIPPY Patient and Public Involvement and Engagement group; comprising a diverse group of younger patients with lived experience of THR. Members contributed their expertise and knowledge to inform and refine areas of research focus and aspects of study design and will contribute to dissemination of study results.

### Data collection and analysis

20 consultant hip surgeons participated in a semistructured online or telephone interview between May and November 2024. Interviews were conducted by CP (female, PhD, experienced qualitative health services researcher), previously unknown to the participants. A verbal consent process was completed before the interview began including for audio-recording and publication of anonymised quotations. A topic guide was developed with input from academic and clinical advisors of the HIPPY research team (online supplemental file 1), and used flexibly to ensure coverage of key topics including preferred fixation choices relating to younger/older patient groups and perceived benefits or disadvantages, while also allowing exploration and clarification of additional topics in detail as they arose. Post-interview CP wrote a short reflexive summary. Interviews ranged from 30 to 90 min, were transcribed verbatim, anonymised and checked against the original audio-recording (transcripts were not returned to participants). The final sample comprised surgeons representing 15 English NHS sites, two Scottish health boards and one private surgeon. Years in practice at consultant level were: 1–5 years: 5; 6–10 years: 7; 11–20 years: 4; over 20 years: 4. Participants’ survey responses identified the average number of hip replacements performed annually as 91 (volume data were unavailable for three surgeons). In ‘younger’ patients (broadly conceived as those under 70) seven participants primarily used cemented and hybrid constructs; and 13 used uncemented and hybrid constructs.

We conducted an inductive analysis to generate themes from the data, including repeated close reading of interview transcripts and constant comparison of data both ‘within’ and across cases.^19 20^ Transcripts were inductively coded in NVivo data management software. Codes were then compared and refined; coded data were grouped into emerging thematic categories and descriptive summaries produced. Interview data generated with surgeons who primarily used cemented and hybrid (cemented/hybrid preference group) and those who used uncemented and hybrid fixations (uncemented/hybrid preference group) in patients under 70 years were compared with the aim of identifying commonalities or differences in features of decision-making about fixation. CP met regularly with AM (PhD, experienced qualitative health services researcher) to discuss emerging categories and to further refine themes. Participant checking was not used. Data collection and analysis took place concurrently and interviews ceased when data saturation was achieved.^18^

## Results

**Figure 2:**
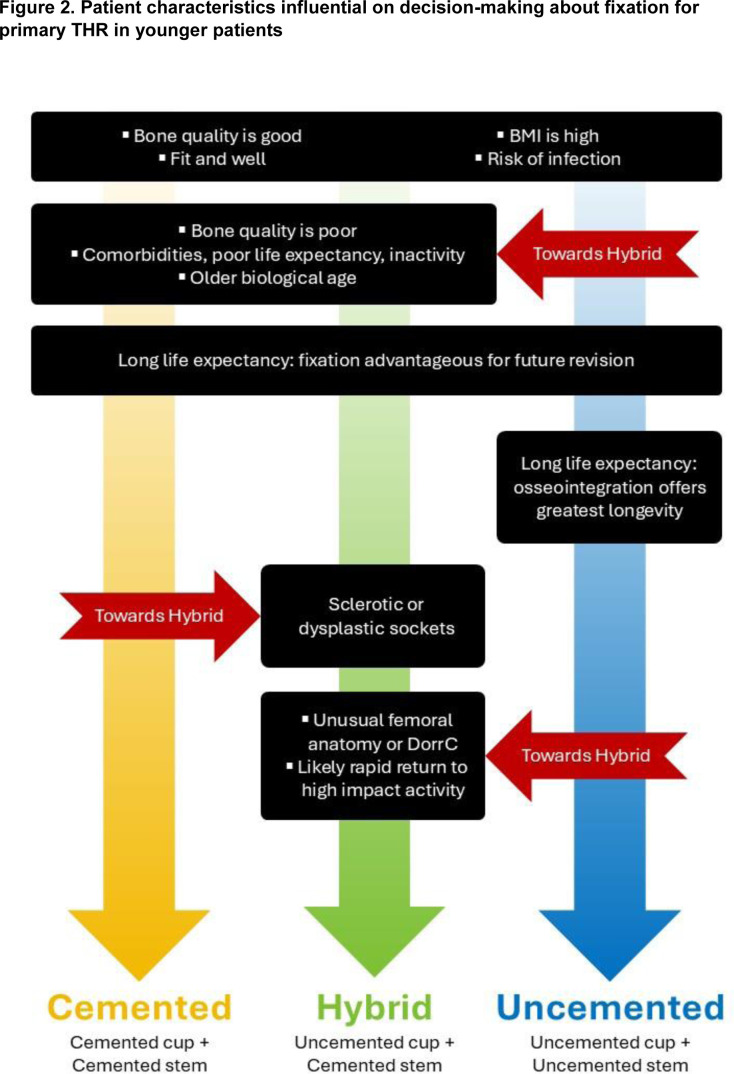
Patient characteristics influential on decision-making about fixation for primary THR in younger patients. BMI, body mass index; THR, total hip replacement.

The final sample of 20 participating surgeons comprised seven surgeons that self-identified as primarily ‘choosing’ between either ‘cemented and hybrid’ fixations (meaning they rarely/never used uncemented stems in young patients) and 13 surgeons who primarily chose between ‘uncemented and hybrid’ fixations (meaning they rarely used cemented cups in young patients). Each surgeon provided a detailed explanation for their decision-making which included maximising the likelihood of achieving a well-reconstructed and long-lasting hip, comprising stable implant components; minimising the risk of complications or early revision; and/or to ‘ease’ a revision in the future. The sample comprised surgeons who favoured cemented, hybrid or uncemented implant fixation types respectively when performing primary elective THR for uncomplicated patients aged under 70 years due to osteoarthritis. An overarching theme of community equipoise was identified in relation to the superiority of one fixation choice over another. In total, we identified six themes describing influences on surgeons’ decision-making about fixation choice; ‘patient characteristics’ (see figure 2); ‘patient preferences and implant availability’; ‘training and establishing a fixation skillset’; ‘formal evidence of outcomes’, ‘personal outcomes and accumulated clinical experience’ and ‘secondary benefits of surgical speed and cost-effectiveness’.

### Patient characteristics

Surgeons’ descriptions of the circumstances under which they used one or other of their preferred fixations were highly revealing of patient characteristics that influenced decision-making. Bone quality, age, comorbidities, activity levels, life expectancy and anatomy/morphology were commonly influential on the fixation rationales of surgeons regardless of whether they used ‘cemented and hybrid’ or ‘uncemented and hybrid’ fixation methods.

#### Younger age: long life expectancy, higher activity demands

All surgeons held the view that patients who were likely to live longer and/or make greater demands of their hip implant were more likely to need a revision. Surgeons who preferred uncemented/hybrid fixations commonly suggested that osseointegration was most likely to achieve greatest implant longevity and minimise the risk of aseptic loosening over longer periods.

0030 I am quite keen on the idea of biological fixation…in terms of wanting to get osseointegration and hopefully fewer complications in the future in terms of aseptic loosening [Uncemented/hybrid surgeon]

This was not universal, however, as another surgeon in the group suggested that those who lived longer were more likely to need a revision and therefore using hybrid fixation in patients under 60 would have the advantage of being less destructive to revise than an uncemented stem.

Some surgeons described a preference for uncemented cups in younger, more active patients because they allowed larger head sizes to be used, which gave patients more range of movement and reduced the risk of dislocation, while also preserving bone, as the use of a larger head size with a cemented cup would require more reaming (removal of bone to secure the cup).

0209 I have better head size options going up to 36 with the hybrid fixation, which I don’t have with the cemented option […] a lot of the younger patients do more activities and more activities often… I think the risk of dislocation increases if you are using a smaller head [Uncemented/hybrid surgeon]

More generally, surgeons in both the cemented/hybrid and uncemented/hybrid preference groups described uncemented cups as having technical advantages for younger adult patients; including multiple bearing/liner combinations and the ability in future to ‘swap’ to new liners while retaining the well-fixed cup. Uncemented cups were therefore perceived to have a ‘futureproofing’ advantage.

0031 different options in terms of bearings to go for…at primary surgery and revision surgery as well…[uncemented] cups have options for dual mobility or constrained liners [Uncemented/hybrid surgeon]

Rapid return to high activity: While many surgeons in the uncemented/hybrid preferring group prioritised uncemented constructs for young, well and active people, it was noteworthy that four of these surgeons nevertheless reported that a patient’s likely rapid return to high-impact activity after THR (running, ‘extreme’ sport, self-employed physical occupations, immediate high loading due to body mass index (BMI)) may influence them to use a hybrid construct, to reduce the risk that high impact would disrupt bone on-growth to the uncemented stem during the early recovery period.

0045 I would be happy for someone to start running sooner with a hybrid than an uncemented implant [Uncemented/hybrid surgeon]

#### Bone quality and holistic assessments of ‘age*’*

Bone quality is an important influence on surgeons in both fixation preference groups; uncemented/hybrid surgeons commonly described the presence of ‘good’, strong bone as fundamental to their use of uncemented constructs, whereas cemented/hybrid surgeons used their preferred fixations in both strong and weak bone. In the presence of weak bone, nearly all surgeons chose to fix their stems using cement to reduce the risk of intraoperative periprosthetic fracture and to achieve a good initial ‘hold’. However, while cemented/hybrid surgeons commonly used a cemented cup as well as cemented stem (cemented fixation) in younger patients with perceived poor bone quality, by comparison uncemented/hybrid surgeons commonly paired a cemented stem with an uncemented cup (hybrid fixation), possibly augmented with screws (although some specified that in specific cases of young people with rheumatoid arthritis or cancer they would also cement the cup). Therefore, poor bone quality influenced surgeons to use cemented stems but not all surgeons necessarily used cement on both sides of the construct.

0034 most young patients have good bone, but obviously if didn’t have good bone [would use a cemented cup] [Cemented/hybrid surgeon]

0208 whether the bone quality is good or poor [uncemented cup] works [Uncemented/hybrid surgeon]

In addition to visual appraisal of X-rays, surgeons described how bone quality diminished with advancing chronological age and all cemented/hybrid surgeons described using cemented constructs for the majority of THRs from about 70–75 years onwards. Some uncemented/hybrid surgeons described specific age points at which they would likely use more cemented stems (hybrids) (although these age points varied by surgeon from 65 to 75 years). However, other surgeons were explicit that chronological age should be considered as one aspect of a more holistic assessment of ‘biological or physiological’ age, as those who are considered ‘biologically older’ are likely to have poorer bone quality. Among uncemented/hybrid preferring surgeons, physiological age often appeared more important than chronological age, and a number of surgeons stated they would continue to use uncemented fixations in (male) patients over 70 if they had good bone quality.

0028: When they’re above 70, I’ll think as to whether I should be doing a hybrid, and if they have got good bone stock, and active, and not lots of medical comorbidities, I would then choose an uncemented—fully uncemented—construct. [Uncemented/hybrid surgeon]

0026: it’s more dependent on the patient’s physiological status than their age [Uncemented/hybrid surgeon]

Collectively, surgeons described diagnoses of osteoporosis, Paget’s disease, metabolic bone disease, endocrine problems, inflammatory arthritis, Chronic Obstructive Pulmonary Disease, steroid use and being a smoker, as well as inactivity and immobility, as likely indicators of poor bone quality. Female sex also appeared a proxy for poor bone quality as surgeons in both preference groups described using cemented or hybrid constructs respectively at an earlier age for women (aged anywhere from 60 to 75 years), than men due to the likely effects of the menopause.

0047 the men 60 and below would get an uncemented hip, women I tend to do hybrid because they’ve gone through the menopause [Uncemented/hybrid surgeon]

0035 I probably do more cemented cups in the postmenopausal females [Cemented/hybrid surgeon]

#### Dense/sclerotic bone

Among cemented/hybrid preferring surgeons, dense or sclerotic bone in the acetabulum (described as common but not exclusive to young, fit men with physical occupations) commonly influenced use of uncemented cups (hybrid construct).

0206 the younger males with OA they’ve got much harder bone, it’s much more difficult to do a decent cemented cup in that age group…although I have done it in plenty [Cemented/hybrid surgeon]

Sclerotic bone required extensive reaming to achieve the cancellous bone interface necessary to achieve cement interdigitation, whereas uncemented cups were perceived to get better fixation without having to remove excessive bone.

#### Dorr classifications

Among uncemented/hybrid preferring surgeons Dorr C femurs (those with a wide canal) were agreed to require a cemented stem (hybrid construct), however there was variation between these surgeons in reported use of uncemented or hybrid fixation for Dorr A and Dorr B femurs.^21^

#### Femoral and acetabular anatomy/morphology

Uncemented/hybrid surgeons described femoral anatomical or morphological variants as a key influence on their decision to use a cemented stem (hybrid construct), if they believed these variations would prevent them from achieving a good fit or the correct positioning of an uncemented stem. Surgeons commonly reported that for version abnormalities, dysplasia, previous osteotomy and wide femoral intramedullary canals, cemented stems were preferable because their position in the cement could be flexibly and precisely ‘finessed’ to optimise hip reconstruction; and further surgeons commonly stressed that ‘not all femurs fit an uncemented stem’. However, anatomical variants did not always require hybrid fixation as uncemented/hybrid preferring surgeons described that some anatomical issues could also be resolved by use of modular uncemented implants, custom uncemented stems or willingness to ream more extensively.

0031 if I’ve got somebody with dysplasia and very marked version abnormalities of the femur, then I have used cemented stems in younger patients [Uncemented/hybrid surgeon]

Cemented/hybrid surgeons described that shallow or dysplastic sockets influenced them to use uncemented cups (hybrid construct). Non-hemispherical sockets were considered unsuited to ‘contain the cement’, and cemented cups were deemed to require complete coverage by bone; therefore, surgeons described achieving a better ‘fit’ with uncemented cups in these cases.

0207 With an uncemented cup, you can leave it overhanging. Whereas if you do that with a cemented cup it tends to fail [Cemented/hybrid surgeon]

#### BMI, risk of infection, likely need for future revision due to long life expectancy

Surgeons in both fixation preference groups described BMI, risk of infection and likelihood of future revision as influential on their decision-making. However, there was variability, rather than congruence of fixation methods used for these characteristics. Higher BMI was cited by surgeons as a reason to use cemented, hybrid and uncemented fixation, respectively; with surgeons commonly reporting that their chosen fixation would achieve better placement of components and consequent reconstruction of the hip. Increased risk of infection (linked to organ transplant, immunosuppressant or steroid use, presence of inflammatory disorders, diabetes, recurrent urinary tract infections or previous infection) influenced some surgeons to use cemented or hybrid fixation as it allowed them the option to use antibiotic-eluting cement. However, another surgeon used uncemented components to avoid the need for future cement removal which would be destructive of bone.

The increased likelihood that younger patients would need a future revision (due to longer life expectancy) influenced several surgeons to use a fixation method they believed would ease a future revision; however, surgeons described uncemented stems, cemented stems and uncemented cups respectively to be ‘easier’ to revise. Cemented stems were reported to be easy to remove and replace, thereby avoiding the bone destruction involved in extracting a ‘well grown in’ uncemented stem. Uncemented stems (and cups) avoided the need for cement removal (and associated bone destruction).

### Patient preferences and implant availability

A small number of surgeons gave some patients a degree of choice in the fixation used, or the option of a ceramic-on-ceramic bearing (necessitating an uncemented cup). When patients required a primary THR on their opposite hip, surgeons (and reportedly patients) commonly reported a preference for matching the fixation to the existing THR, assuming the initial hip had performed well. Some surgeons reported that availability or model of implants stocked within the Trust could on occasion influence fixation choices, either when components were out of stock (as long as they were confident that patient outcome would be unaffected), or if they perceived that a stocked implant component was of unproven quality in comparison to another available implant.

### Training and establishing a fixation skillset

Surgeons in both groups reported the significant influence of their training on the fixations they used. Although most surgeons reported being exposed to ‘all three fixations’ at some timepoint during their training, many also described that their training had been oriented towards attaining either a cemented/hybrid or uncemented/hybrid fixation skillset and that they had acquired an accompanying rationale and conviction in those fixation methods (rather than becoming equally proficient in all three methods). Although a small number of surgeons in the uncemented/hybrid group reported that they had sought to maintain a skillset in all three fixations to give them greater versatility and flexibility, they reported only using cemented cups in the young in specific and rare exceptions (see above). The interviews revealed that many UK hip units and geographic regions had ‘fixation traditions’ (meaning a tradition of using two of three fixations) and eight surgeons reported that they were currently working in (separate) units in which only cemented/hybrid or uncemented/hybrid fixations were used for younger people. Because arthroplasty surgeons receive training via a ‘mentored apprenticeship’ they could be trained in units that either used cemented and hybrid or uncemented and hybrid fixations and therefore the fixation skills they carried forward in their practice could be limited to two of the three methods. It was commonly reported that training in units that practised cemented/hybrid or uncemented/hybrid fixation would limit exposure to uncemented stem or cemented cup fixation skills respectively, and lead to a decline of cemented cup skills, due to lack of exposure to the technique.

0032 in our region we do a lot of cemented…if you go towards London you could go through your entire training and not see a cemented hip—either stem or cup [Cemented/hybrid surgeon]

0030 almost all my training was in uncemented arthroplasty [Uncemented/hybrid surgeon]

One surgeon suggested that units with a fixation tradition may reinforce this tradition by only employing those with the same fixation skillset, which excludes surgeons with a different fixation philosophy. Alternatively, surgeons could be employed and trained up in the favoured fixation methods of the hip unit, despite the initial emphasis of their training. Some surgeons who had trained in all three fixations had aligned their practice with the specific tradition within the unit they joined as a consultant. It appears therefore that surgeons might choose to join a unit which matches their fixation skills or might prioritise certain fixation methods to fit into the tradition of the hip unit. Therefore, unit fixation tradition could exert influence on both the skills attained during training and those continued in clinical practice over time.

0207 if I’d trained in a … different part of the country or was practising in a different hospital my practice would undoubtedly be different because you have to practise what feels comfortable within the group you’re working in. As long as they’ve got good outcomes [Cemented/hybrid surgeon]

0047 I’ve only really started doing uncemented hips since becoming a consultant [Uncemented/hybrid surgeon]

Interviews further revealed that surgeons refined their skills in fixation methods through continued practice over time (the learning curve). Depending on their clinical practice and training, surgeons often developed skills in two fixation methods over three and their confidence in those methods was reinforced through continued use (as long as their outcomes were good). In contrast use of fixations or components with which surgeons were less familiar would involve ‘a learning curve’ during which outcomes would likely be affected, until familiarity and competence was established in the new technique.

0030 the worst thing for me, for my patients, and indeed for the results of hip arthroplasty as a whole is if you suddenly get a load of people who are starting to do something that they haven’t done before, because the early results will be poor [Uncemented/hybrid surgeon]

0027 we use what we are used to using [Cemented/hybrid surgeon]

Many surgeons perceived that good surgical outcomes were fundamentally linked to the achievement of skill in a modest number of practices and that poorer outcomes were associated with frequent changes of fixation method or inconsistent use of a variety of techniques or implant components.

0207 being consistently good- a bit like sport, isn’t it? You’re much better concentrating on one sport [Cemented/hybrid surgeon]

Surgeons also described their fixation choices being influenced by senior colleagues and peers. Some who initially trained in cemented and hybrid fixation described how they later retrained in uncemented fixation after observing colleagues use this method for younger patients. Others described how leaders in the field or peers, who made similar choices, gave them confidence to change practice. Conversely, if their own fixation decisions differed from most of their colleagues, this could generate uncertainty.

0036 [if most of my colleagues don’t do cemented cups] you start getting doubts in yourself [Cemented/hybrid surgeon]

Opinions shared in formal multidisciplinary team meetings were also described as highly influential.

### Formal evidence of outcomes

Surgeons in both fixation groups reported being influenced by formal evidence from clinical research studies (often cohort studies led by individual hip units) and evidence of good long-term results from UK NJR analyses; specifically focused on the low risk of revision for the fixations (and implant/model types) they used. It was common for surgeons in both fixation groups to describe the equivalence of revision estimates between (high quality) cemented and uncemented stems; and uncemented/hybrid surgeons commonly reported that uncemented stem outcomes were comparable to cemented stems in terms of low revision risk. One surgeon also reported evidence of better patient ‘experience’ associated with use of uncemented constructs.

0027 both systems have great outcomes…the overall survival of the implants is very much equivalent [Cemented/hybrid surgeon]

0209 [Uncemented constructs] have just as good results as cemented in people under the age of 65, and similar results between 75 and 65 [Uncemented/hybrid surgeon]

Therefore, although some uncemented/hybrid surgeons preferred uncemented fixation in younger people, perceiving that in theory bone ongrowth gave greater longevity and a reduced risk of aseptic loosening, they acknowledged there was currently no formal evidence for this.

0047 having an implant…the bone has grown into and it’s locked as part of the body, theoretically should give greater longevity [Uncemented/hybrid surgeon]

0045 if uncemented goes right, then the longevity might be higher, even though that hasn’t been shown [Uncemented/hybrid surgeon]

In relation to uncemented cups however, uncemented/hybrid surgeons reported being influenced by NJR (and other registry) analyses showing that uncemented cups had superior survivorship (longevity) in both primary and revision hip replacements when compared with cemented cups. There were also surgeons in the cemented/hybrid group who reported increasing their use of hybrid fixations in younger people (in part) due to NJR evidence showing good outcomes for uncemented cups.

0034 the uncemented cup data on the NJR […] relatively recently demonstrated ‘as good’ outcomes, and possibly slightly better, for younger people [Cemented/hybrid surgeon]

048 looking through the joint registries, the uncemented cups tend to have the longest track record overall [Uncemented/hybrid surgeon]

Evidence relating to periprosthetic fracture risk was also influential within each preference group. Cemented/hybrid surgeons reported evidence of lower early periprosthetic fracture risk associated with use of cemented stems. Uncemented/hybrid surgeons commonly referred to recent evidence that uncemented stems (with a collar) reduced periprosthetic fracture risk compared with polished tapered cemented stems, and that fractures linked to cemented stems had been obscured in registries due to a focus on revision rather than reoperation. Others perceived that higher periprosthetic fracture risk associated with uncemented constructs could be mitigated by surgeon experience and sensible patient selection.

0206 with cemented hips there’s a lower risk of revision in the first two years…particularly in relation to periprosthetic fracture [Cemented/hybrid surgeon]

0029 higher fracture rate…in patients who have uncemented hips…is down to poor technique, poor selection of the patient, rather than…the implant [Uncemented/hybrid surgeon]

Surgeons in both preference groups made claims to formal evidence to support their fixation choices, and further could interpret the same NJR evidence as supportive of their differing stem fixation choices. Similarly, some cemented/hybrid surgeons had continued to use cemented cups in younger people, perceiving NJR evidence relating to uncemented cup survivorship in under 55s to be ambivalent. Interviews identified that the fixation ‘evidence base’ comprised multiple sources, and that contesting interpretations of formal evidence could co-exist. At times surgeons explicitly acknowledged the shifting or indefinite nature of evidence.

0043 It’s a little bit like politics to me. You can twist the data to say whatever you want [Uncemented/hybrid surgeon]

### ‘In my hands’: personal outcomes and accumulated clinical experience

In addition to formal evidence, surgeons in both fixation preference groups referred to more ‘personal’ evidence, based on experience and observation in clinical practice, that their fixation choices performed well and resulted in good outcomes for their patients.

0036 doesn’t matter if you’re young or old…decent cement and a polished tapered stem, they last [Cemented/hybrid surgeon]

0029 if you look at uncemented stems done properly, 20 years down the line, they look identical to the day they were put in [Uncemented/hybrid surgeon]

Surgeons in the cemented/hybrid group had also experienced excellent performance of cemented cups. However, it was common for uncemented/hybrid surgeons to contrast personal experiences of excellent uncemented cup performance with failures or loosening of cemented cups which had influenced their use of uncemented cups in all young patients.

0030 we’ve moved away from cemented sockets […there were] issues with socket loosening around the 10-12 year mark [Uncemented/hybrid surgeon]

Surgeons in both groups also referred to technical advantages ‘inherent’ in the fixations they used as influencing or reinforcing their fixation decisions. Surgeons in the cemented/hybrid group reported cemented stems allowed maximum control and flexibility of leg length, offset and version (uncemented/hybrid preferring surgeons utilised these benefits in cases of unusual anatomy). Many surgeons within the uncemented/hybrid group (and some in the cemented/hybrid group) reported uncemented cups to be easier to correctly orient or to reposition intraoperatively; thereby optimising reconstruction of the hip.

Surgeons often referred to their NJR consultant-level report which evidenced that they were personally achieving good outcomes using their chosen fixations and by extension that they were highly skilled in their preferred fixation skillsets which reinforced their continued use:

0207 I’ve been in the fortunate position of being an underlier for my hip replacements… you’ve not really got anywhere to go for improvement [Cemented/hybrid surgeon]

0028 I can back it up, and I’m not an outlier in terms of what I’m doing […] we know what works in our hands [Uncemented/hybrid surgeon]

In contrast, several surgeons also reported instances where personal evidence of poor outcomes/component failures, and concern about becoming ‘an outlier’, had influenced them to make changes to their fixation decisions. Experiential evidence gained through observation and practice was therefore often given primacy over more ‘formal’ evidence sources.

### Surgical speed and cost-effectiveness as secondary benefits

Time saved was widely acknowledged to be an advantage of uncemented fixation (or components), however, participating surgeons were careful to note that this was not the main influence on their decision-making, instead emphasising that good patient outcomes came first.

0034 there is a slight advantage of [uncemented cup] being a bit quicker…same outcomes and a bit quicker is better [Cemented/hybrid surgeon]

Similarly, surgeons commonly reported cost-effectiveness to be a secondary benefit of the fixation choices they made for younger adult patients rather than a main driving influence on their decision-making, emphasising that cost-effectiveness for the NHS was prioritised only after good or equivalent fixation outcomes were established by formal evidence.

0207 I don’t think it changes my practice, but we all have a duty to not excessively spend money if something is as good if not better and it’s cheaper [Cemented/hybrid surgeon]

0031 in terms of the outcomes…it doesn’t seem to make much difference [between uncemented and cemented stems]… therefore using something with a potentially lower cost is beneficial [Uncemented/hybrid surgeon]

Surgeons in both fixation groups perceived that their fixation choices respectively offered ‘best’ value to the NHS (often linked to the success of Trust/regional procurement processes). Some uncemented/hybrid surgeons linked greater cost-effectiveness to surgical time efficiency which enabled increased volume of cases in the health system (reduced infection risk was also a benefit of shorter surgical time); however, some cemented/hybrid surgeons disputed whether time saved would equate to inclusion of additional patients on a mixed practice NHS list. Surgeons in both fixation groups did however perceive that increased surgical speed was a likely influence on ‘some’ surgeons who used uncemented constructs, particularly those with high volume lists or undertaking THR privately for whom they believed poorer productivity might have economic consequences.

0047 [people] put in an uncemented stem, particularly in the private sector because it means they can get more work done on a list. You know, it does save you time […] I think there is an element of quickness for some surgeons, I don’t think it’s for everyone [Uncemented/hybrid surgeon]

## Discussion

This qualitative in-depth exploration of surgeons’ decision-making about fixation in patients aged under 70 years may be the first to have compared decision-making related to different surgeons’ fixation choices which has enabled the identification of key influences on surgeons’ fixation choice. We identified that there were surgeons who prioritised cemented, hybrid and uncemented fixation respectively for young and ‘uncomplicated’ patients which demonstrates an overall community equipoise regarding which method is clinically superior.^22^ All surgeons in our sample described detailed rationales for their fixation decisions and these often-shared features with others both within and across fixation preference groups. All surgeons described being influenced by patient characteristics when deciding between cemented and hybrid or uncemented and hybrid fixation methods with the primary aim to achieve a well-constructed and long-lasting hip and to reduce the risk of complications should a future revision be needed. Poor bone quality was the patient characteristic around which most surgeons agreed about a single ‘best’ fixation; specifically, use of cemented stems. However, fixation method used in the acetabulum varied when bone was poor. This finding echoes that of Karia *et al*,^23^ of a lack of consensus about optimal acetabular fixation method in patients with poor bone quality. Our findings expand an understanding of the holistic way in which surgeons make assessments about bone quality; identifying the importance of ‘biological/physiological age’, which may incorporate assessment of chronological age alongside health, activity, life expectancy (and female sex). These findings lend further explanatory context to recent research which identifies age and activity level as the most influential patient characteristics on surgeons’ fixation choice.^15^ We also identified a general agreement around anatomical variations on the femoral and acetabular side; for which many surgeons favoured use of cemented stems and uncemented cups respectively, and that sclerotic bone also influenced uncemented cup use.^24^ However, there were also exceptions as types of uncemented stem could be used to address variations in femoral anatomy or sclerotic bone reamed to accommodate a cemented socket. High BMI, risk of infection and likely need for a future revision due to long life expectancy influenced different surgeons to use different fixations. Their decisions were underpinned by rational explanations that specific fixation choices would enable a ‘better’ reconstruction, minimise complication risk or ‘ease’ any required revision. Patient’s likely rapid return to high activity influenced some surgeons who generally prioritised uncemented fixation in young patients to use hybrid fixation.

Our study identified that training and establishment of a fixation skillset were important upstream influences on fixation decision-making and problematises the claim that ‘most surgeons in the UK are trained to use both cemented and uncemented prostheses’.^25^ Interviews revealed that geographical influence or unit fixation traditions may delineate the fixation skillsets into which surgeons are trained and continue to practice. This is reminiscent of the ‘organisational knowledge’, identified by Grove *et al*, which shapes the perspectives of clinicians working in an organisation…and influences the behaviour of its members’.^10^ Further, surgeons establish familiarity and competence in specific fixation skillsets through continued practice over time; and achievement of good patient outcomes in surgery was perceived to be fundamentally linked to individual high-volume practice in a restricted number of surgical techniques, allowing surgeons to become highly skilled.^26^ We identified the particular importance of the NJR consultant level report as a key source of evidence of surgeons’ personal outcomes and likely to reinforce fixation choice if outcomes were good.^27^ Recent research identifies that surgeons who used more brands of implant had increased odds of being an outlier; therefore, surgeons were unlikely to deviate from their established fixation skillset without good reason.^28^ There is a well-established volume-outcome relationship in surgery and the related ‘learning curve’ presents a unique challenge to trial design.^29^

Previous research has identified the ‘low influence’ of formal codified knowledge (including independent peer-reviewed literature), the consequent impediment this poses to evidence-based decision-making; and that high importance is placed on experiential knowledge in orthopaedic practice more generally.^10 11 30 31^ While we identified that surgeons’ accumulated clinical experience of good fixation performance (and personal good outcomes) was an important influence and for some surgeons of parallel importance to ‘formal evidence’ in influencing fixation decisions, we also identified in common with others that surgeons frequently cite formal evidence (particularly relating to revision estimates and periprosthetic fracture risk) as influential on (and justificatory of) their fixation decision-making.^32^ Formal evidence of the superior longevity of uncemented cups was widely acknowledged and may partially explain increased (and increasing) use of uncemented and hybrid fixations in younger people, in addition to their perceived technical benefits.^1^ However, we also identified that the fixation evidence base comprised multiple sources, and that interpretation of formal evidence, such as NJR outcome data, varied as surgeons referenced the same evidence source as influential for a variety of fixation choices. Similarly, previous orthopaedic studies have observed that the accumulation of scientific data may not result in movement towards ‘an agreed scientific consensus’.^31^ Formal evidence identifying comparable outcomes between cemented and uncemented stem fixations was commonly reported, thereby legitimising the choice of surgeons for both cemented and uncemented fixations. The theoretical likelihood that uncemented constructs offer greatest longevity in the long-term may also be an important explanatory factor for the use of uncemented fixation in younger patients.^33^ Our findings further identify a potentially important balance between the influence of ‘formal evidence’ and individual surgeons’ established skillset and personal outcomes; we question whether evidenced practices that can be accommodated within the existing skillset may have a greater likelihood of adoption.

The influence of peers and respected colleagues on surgical decision making is well established.^10 11 30 32^ However, our identification of traditions of fixation practice necessitates consideration of the extent to which respected colleagues may be located ‘within’ the same fixation traditions. Increased surgical speed of uncemented components may be influential on decision-making for some surgeons but not for others. The previously evidenced higher cost of uncemented components also appeared less influential as implant cost-effectiveness was considered secondary to outcomes when making fixation decisions.^4 34^ Local availability of implants could, however, affect decision-making.

Qualitative methods enabled us to explore features of decision-making about fixation in a sample varied by years of experience across fixation methods, annual THR volume and geographical location. We acknowledge that recruitment of consultant hip surgeons through membership of the British Hip Society and via an ‘expression of interest’ in an online survey may have recruited a particularly ‘research oriented’ sample. This particular sample might have prioritised formal evidence and patient characteristics as important influences on decision-making about fixation over institutional or habitual factors. Interviews further comprise accounts that are socially and contextually produced with responses likely to emphasise competence, particularly when describing professional practice. We also acknowledge that inclusion of only UK-practising consultant surgeons may limit transferability to other healthcare systems where implant availability, procurement processes and registry processes may differ.

Fixation traditions may exert influence on fixation skills gained during training and continued in clinical practice. Over time surgeons may establish competence in specific fixation skillsets, to which likelihood of good outcomes is inextricably linked, likely reinforcing continued use of those methods, particularly when outcomes are confirmed by the NJR consultant level report. Formal evidence of fixation outcomes was influential but also comprised multiple sources and differing interpretations, rather than facilitating overall agreement relating to ‘best’ fixation method for younger patients. Many patient characteristics (young and uncomplicated, poor bone in the acetabulum, high BMI, rapid return to high activity, risk of infection, likely need for revision) influenced differing fixation decisions between surgeons. Our study has identified an overarching ‘community equipoise’ across the UK hip surgeon community relating to which fixation method is superior for young, uncomplicated patients or relative to specific patient characteristics.^22^ These findings demonstrate the need for an RCT of fixation methods, while highlighting patient characteristics around which randomisation may be problematic.

## Supplementary material

10.1136/bmjopen-2026-118009 online supplemental file 1

## Data Availability

Data are available on reasonable request.
