# Supplementary material for: What influences UK-practising consultant hip surgeons’ decision-making about implant fixation? An interview study
Source: BMJ Open. 2026 Jul 10;16(7):e118009. doi: 10.1136/bmjopen-2026-118009 (PMC13358266; doi:10.1136/bmjopen-2026-118009)
Supplement: online supplemental file 1 [file bmjopen-16-7-s001.docx]

**HIPPY Study – surgeon topic guide 110823**

**Opening**

How many years have you been doing primary elective Total Hip Replacement as a consultant?

**Exploring decision making about fixation**

Do you have a preferred / most used fixation choice for your NHS-funded patients aged under 70 who require THR due to osteoarthritis? (cemented / uncemented / hybrid / reverse hybrid)

Why do you favour that choice of fixation for the majority? What are the benefits of that fixation? Are there any disadvantages?/risks?

Does your choice of fixation differ for your NHS-funded patients aged 70 or older who need Total Hip Replacement due to osteoarthritis? Why is that?

Are there circumstances under which you wouldn’t use your preferred* fixation choice (**insert as applicable cemented/uncemented/hybrid/ reverse hybrid**) for patients aged under 70 and what would be the reason(s) for this?

What outcomes do you think are most important after a THR? (Pain relief, mobility, return to work, implant longevity) – does the importance of a particular outcome influence which implant fixation you choose?

In patients aged under 70 - what characteristics of the patient do you take into consideration when choosing fixation/implant type (e.g. age, medical history, expected activity levels). Are there key patient characteristics that you think about more than anything else?

Are there other things that you take into consideration when decision making about fixation, that are not about the patient? (e.g. length of time in theatre/easier to revise/implant revision rate/policies and guidelines/published evidence/peers and mentorship) – how much weight do these elements carry in your decision?

Do you personally audit your practice?

Have you ever changed your fixation choice for patients aged under 70 who require Total Hip Replacement due to osteoarthritis (*e.g. cemented to hybrid/hybrid to uncemented*)? Could you tell me why this change occurred?

Are there particular models/brands of implant/construct that you use most often in patients aged under 70? Why do you favour these ones? (e.g. training/ODEP rating/rep support)

Do you use different fixation/implants in your private patients under 70, compared to your NHS-funded patients under 70 who need primary Total Hip Replacement due to OA? If so, why? Why not.

**Procurement of implants**

Are you involved in the procurement process for hip replacement implants at your Trust? (If you are, is that as a lead for the process or as a contributor to the process?, if not, who does lead the procurement process for hip replacement implants at your Trust?)

In your time as a surgeon doing hip replacements, has there been a procurement process run in your Trust to decide what hip replacement implants are available?

- What form does that implant procurement process take?

Who makes the ultimate decision as to what implants are available to you?

Do you feel that your input to the procurement process does affect the outcome?

Is evidence of outcomes for the hip implant options used as part of the procurement process?

What factors do influence the decision on what hip implants are available?

Have you ever in your practice wanted to use a particular implant but not been able to?

**Exploring information given to patients about fixation/ implants:**

Are your NHS patients involved in the decision making about which implant is used for their Total Hip Replacement?

What information do you provide to patients about the fixation/implant you intend to use? (fixation, brand, model, bearing surface, why this is significant?)

Do you provide information about risks/benefits of different fixation/ implants?

What do you think patients should know about the fixation/implant they are being given? Do they get a choice?

**Acceptability of the HIPPY RCT**

*Within the HIPPY research programme we plan to undertake a three-group clinical trial in which patients under 70 requiring primary Total Hip Replacement due to osteoarthritis will be randomised to receive an uncemented, cemented or hybrid implant.*

Would you potentially be willing to randomize your patients in this trial to those three groups? (If not, why not?)

Would you be willing to randomize them to just two of the three groups – which groups?

What proportion of your patients aged under 70 would you be willing to randomize?

Which patients would you not be willing to randomize?

The primary outcome will be revision or not at 10 years after surgery. Which of the proposed secondary outcomes do you think are the most important? (show list of proposed secondary outcome measures)

If the trial demonstrated a clear benefit for a fixation choice that differed from your preferred current practice, would it change your practice?

How challenging would it be for you to potentially change your practice, based on the results of the trial? What would the challenges be? How could these be overcome?

**Closing section**

Is there anything you would like to add or anything you wish to talk about that we haven’t covered already?
