# Supplementary material for: What influences UK-practising consultant hip surgeons’ decision-making about implant fixation? An interview study
Source: BMJ Open. 2026 Jul 10;16(7):e118009. doi: 10.1136/bmjopen-2026-118009 (PMC13358266; doi:10.1136/bmjopen-2026-118009)
Supplement: Supplementary file 2 [file bmjopen-2026-118009.reviewer_comments.pdf]

PEER REVIEW HISTORY

BMJ Open publishes all reviews undertaken for accepted manuscripts. Reviewers are asked to complete a checklist review form and are provided with free text boxes to elaborate on their assessment. These free text comments are reproduced below.

ARTICLE DETAILS

Title (Provisional)

What influences UK-practicing consultant hip surgeons' decision-making about implant fixation? An interview study.

Authors

Palmer, Cecily; Baji, Petra; Whitehouse, Michael R; Evans, Jonathan Thomas; Blom, Ashley W; Reed, Mike; Marques, Elsa MR; Moore, Andrew J

VERSION 1 - REVIEW

**Reviewer**	**1**
**Name**	**Sabharwal, Sanjeeve**
**Affiliation**	**Imperial College London, MSk Lab**
**Date**	**30-Mar-2026**
**COI**	**None**

This is a valuable study that will feed into an RCT design and addresses a genuine evidence gap and therefore the findings will be of immediate value. They have mentioned 2 limitations in their discussion - one regarding the selection process of hip surgeons from the BHS, and that "interviews comprise accounts that are socially and contextually produced'. It is worth elaborating on this a bit - specifically that as all interviews were conducted by a single researcher who was previously unknown to participants, and although this ensured consistency, responses may have emphasised evidence based and patient centred reasoning rather than the more habitual or institutional factors that influence everyday decision making. Furthermore, The study included only consultant level surgeons working in NHS or private elective practice in the UK, which limits transferability to other healthcare systems where implant availability, procurement processes, and fixation practices may differ.

**Reviewer**	**2**
**Name**	**Cnudde, Peter**
**Affiliation**	**Swedish Hip Arthroplasty Register**
**Date**	**11-May-2026**

COI **None**

Thank you very much for a well-written manuscript.

I do believe it clearly confirms the reasoning behind implant choice for many surgeons and hopefully will complement the anticipated results of the HIPPY trial.

As this is in my view a thematic analysis, based on the work of Braun and Clarke using the 6 steps approach, having a table/figure with codes and themes might provide further clarification, especially when our specialty gets little exposure to qualitative research.

Congratulations for performing this important work, it might be worthwhile considering this work for internationalisation and use the rationale and methods and find out if there would be different/opposing(?) views in N. America, India, the Scandinavian countries, Australia and New Zealand and/or whether there is a difference in opinion between countries with established registries (and outcome registration) and those who do not. Are there training issues in other countries?

Well done.

VERSION 1 - AUTHOR RESPONSE

Reviewer: 1

Mr. Sanjeeve Sabharwal, Imperial College London

Comments to the Author:

This is a valuable study that will feed into an RCT design and addresses a genuine evidence gap and therefore the findings will be of immediate value. They have mentioned 2 limitations in their discussion - one regarding the selection process of hip surgeons from the BHS, and that "interviews comprise accounts that are socially and contextually produced'. It is worth elaborating on this a bit - specifically that as all interviews were conducted by a single researcher who was previously unknown to participants, and although this ensured consistency, responses may have emphasised evidence based and patient centred reasoning rather than the more habitual or institutional factors that influence everyday decision making. Furthermore, The study included only consultant level surgeons working in NHS or private elective practice in the UK, which limits transferability to other healthcare systems where implant availability, procurement processes, and fixation practices may differ.

We are very grateful to Mr. Sabharwal for his positive comments about the manuscript, and particularly the helpful critique which fairly identifies the need for fuller discussion of the limitations of the study. We have therefore extended the consideration of limitations in both the discussion section (lines 665-675) and the strengths and limitations (related to methods)

section after the abstract (lines 52-64). Specifically we acknowledge that recruitment of 'research oriented' surgeons, as well as the social norm to present competency in interview accounts reporting professional practice may have influenced the extent to which evidence-based and patient-centred factors were prioritised in surgeons' accounts of their decision-making. Although our findings have also identified training and skillset, and the presence of unit/geographical 'fixation traditions' as important habitual or institutional influences that also influence decision-making, we acknowledge that use of observational or ethnographic study designs; or inclusion of less research-oriented surgeons, may broaden understanding of decision-making in practice. We have also included acknowledgement that focussing on UK-practicing surgeons is likely to limit transferability to other healthcare systems (although it does offer potential for cross cultural comparison).

Reviewer: 2
Dr. Peter Cnudde, Swedish Hip Arthroplasty Register, Goteborgs universitet Institutionen for kliniska vetenskaper
Comments to the Author:
Thank you very much for a well-written manuscript.
I do believe it clearly confirms the reasoning behind implant choice for many surgeons and hopefully will complement the anticipated results of the HIPPY trial.
As this is in my view a thematic analysis, based on the work of Braun and Clarke using the 6 steps approach, having a table/figure with codes and themes might provide further clarification, especially when our specialty gets little exposure to qualitative research.
Congratulations for performing this important work, it might be worthwhile considering this work for internationalisation and use the rationale and methods and find out if there would be different/opposing(?) views in N. America, India, the Scandinavian countries, Australia and New Zealand and/or whether there is a difference in opinion between countries with established registries (and outcome registration) and those who do not. Are there training issues in other countries?
Well done.
We are very grateful to Dr Cnudde for his positive comments about the value and clarity of our manuscript. There are many approaches to thematic analysis and while Braun and Clarke's "reflexive thematic analysis" is well known and often cited, it is specific to psychological/social research. In our study we used an approach more in keeping with a health services research design which utilises thematic analysis techniques including repeated close reading, line by line

coding, and constant comparison both within and across data sources as described, and informed by the approach developed by Miles and Huberman, as referenced (19, 20). We have presented final themes, accompanied by illustrative extracts within the manuscript with consideration of permitted word counts; additional information would not change the findings we have presented. We welcome the suggestions relating to potential future expansion of this work to permit cross-cultural exploration and comparison of surgical decision-making related to fixation; and in relation to the presence (or absence) of national registries. These suggestions offer multiple (and intriguing) avenues for further study for which we are grateful.
